# Age-Dependent Safety and Effectiveness of Pridinol Versus NSAIDs in Acute (Low) Back Pain: A Secondary Analysis of the Providence Real-World Study

**DOI:** 10.3390/jcm15134888

**Published:** 2026-06-23

**Authors:** Michael A. Überall, Artur Schikowski, Philipp C. G. Müller-Schwefe

**Affiliations:** 1 Center of Excellence in Health Care Research, Institute of Neurological Sciences—IFNAP, Nordostpark 51, 90411 Nürnberg, Germany; 2 Pain Institute & Center for Medical Education Düsseldorf, Friedrichstraße 13-15, 40217 Düsseldorf, Germany; schikowski@neuro-pain-consult.de; 3 Interdisciplinary Pain and Palliative Care Center Göppingen, Schillerplatz 8/1, 73033 Göppingen, Germany; philipp@mueller-schwefe.com

**Keywords:** acute low back pain, pridinol, nonsteroidal anti-inflammatory drugs, age-related risk, adverse drug reactions, real-world evidence

## Abstract

**Background**: Nonsteroidal anti-inflammatory drugs (NSAIDs) are widely recommended for the treatment of acute (low) back pain, despite modest effectiveness and well-known safety concerns, particularly in older patients. Pridinol is a centrally acting antispasmodic with a mechanism-oriented approach targeting muscle spasm, a key component of acute back pain. While a previous real-world analysis demonstrated a significantly better tolerability and effectiveness of pridinol compared with NSAIDs, age-dependent effects have not yet been systematically evaluated. **Objective**: To assess the age dependency of effectiveness, safety, and tolerability of pridinol versus NSAIDs in patients with acute (low) back pain under real-world conditions, based on already available data. **Methods**: This secondary analysis used propensity score-matched real-world data from the German Pain e-Registry (PROVIDENCE study; EUPAS identifier: 49718). A total of 934 patients with acute (low) back pain treated for four weeks with either pridinol (n = 467) or NSAIDs (n = 467) were stratified by age (<65 vs. ≥65 years). Outcomes included the incidence of adverse drug reactions (ADRs), ADR-related treatment discontinuations, time to ADR occurrence, and clinically meaningful improvement in pain-related disability (≥50% reduction in modified Pain Disability Index). Analyses were performed within and between age strata. **Results**: Overall, ADRs were reported by 9.0% of pridinol-treated patients and 20.8% of NSAID-treated patients (*p* < 0.001). In the pridinol cohort, ADR rates were virtually identical in patients <65 and ≥65 years (8.9% vs. 9.2%; *p* = 0.940). In contrast, NSAID-treated patients showed a pronounced age-related increase in ADR incidence (17.3% vs. 32.1%; *p* < 0.001). ADR-related treatment discontinuation rates under NSAIDs increased markedly with age (5.9% vs. 21.1%; *p* < 0.001), whereas rates under pridinol remained low and age independent (3.1% vs. 4.6%; *p* = 0.447). Gastrointestinal and cardiovascular ADRs were the main contributors to the age-related risk increase under NSAIDs, while corresponding events under pridinol were rare across age groups. Clinically meaningful improvement in pain-related disability was achieved with pridinol/NSAIDs in 91.9/48.0% (<65 years) and 88.1/47.7% (≥65 years; *p* < 0.001 for both). **Conclusions**: Age is a major modifier of NSAID-related risk but not of pridinol tolerability in acute (low) back pain. While NSAID-associated ADRs and treatment discontinuations increase substantially in patients aged 65 years or older, pridinol demonstrates a stable, age-independent safety profile combined with significantly better functional outcomes. These findings suggest that, particularly in older patients, mechanism-oriented alternatives such as pridinol may offer a more favorable benefit–risk profile than NSAIDs.

## 1. Introduction/Background

Acute (low) back pain (a(L)BP) represents one of the most frequent causes for consultations in primary care and emergency medicine and affects up to 80–90% of adults in industrialized countries at least once during their lifetime [1,2,3]. Although spontaneous improvement occurs in a substantial proportion of patients, a considerable number experience persistent pain, functional limitations, or recurrent episodes within weeks to months, which are associated with an increased risk of pain chronification and long-term disability [4,5,6,7,8].

Current national and international guidelines recommend nonsteroidal anti-inflammatory drugs (NSAIDs) as first-line pharmacological treatment for acute low back pain, provided they are used for a limited duration [9,10]. However, evidence from randomized controlled trials and meta-analyses suggests that the analgesic benefit of NSAIDs in this indication is modest and often of questionable clinical relevance [11]. In parallel, NSAIDs are associated with a well-documented spectrum of adverse drug reactions, the incidence and severity of which increase substantially with advancing age. Older patients are particularly vulnerable to NSAID-related gastrointestinal bleeding, cardiovascular events, and renal impairment, due to age-related physiological changes, a higher prevalence of comorbidities, and polypharmacy [12,13,14]. Consequently, age ≥ 65 years is widely recognized as a major clinical risk factor for NSAID therapy, and a large proportion of older patients present with at least one formal contraindication or precaution for their use [12,13].

Given these limitations, alternative pharmacological approaches are of particular interest in patients with acute low back pain of predominantly muscular origin. In the majority of cases, non-specific a(L)BP is caused or accompanied by muscular dysfunctions, characterized by increased muscle tone, abnormal muscle activation, and pain-related movement restriction [15]. In such situations, antispasmodic agents may represent a pathophysiologically plausible treatment option.

Pridinol is a non-benzodiazepine antispasmodic agent exerting its effects primarily via antagonism at muscarinic acetylcholine (M1) receptors and is approved for the treatment of central and peripheral muscle spasms, including those associated with low back pain [16,17]. As with other anticholinergic substances, concerns have been raised regarding its use in older patients, as increasing age is associated with heightened susceptibility to anticholinergic adverse effects such as dry mouth, dizziness, urinary retention, and cognitive impairment [18,19]. However, recent clinical trials, real-world analyses, and a meta-analysis have consistently characterized pridinol as a substance with comparatively low anticholinergic potency and a favorable tolerability profile when used in accordance with recommended dosing regimens, without evidence of a clinically relevant age-related increase in adverse events [20,21,22,23].

The PROVIDENCE study, a retrospective, non-interventional, propensity score-matched dual-cohort analysis based on data from the German Pain e-Registry, demonstrated superior effectiveness and better overall tolerability of pridinol compared with NSAIDs over a four-week treatment period in patients with acute (low) back pain who had insufficient response to self-medication or spontaneous remission [24]. Given the high prevalence of older patients in routine clinical practice and the pronounced age dependency of NSAID-related risks, it is of considerable clinical relevance to explore whether the benefit–risk profile of pridinol and NSAIDs differs according to patient age.

## 2. Study Objective

The objective of the present secondary analysis was to investigate the effectiveness, safety, and tolerability of pridinol versus NSAIDs in patients with a(L)BP stratified by age, comparing patients aged <65 years with those aged ≥65 years, using the existing PROVIDENCE real-world dataset.

## 3. Ethical Considerations

All research reported in this publication was conducted in an ethical and responsible manner and in full compliance with all applicable regulations and standards of good scientific practice. The present work represents a predefined secondary analysis of the PROVIDENCE study and is based exclusively on depersonalized real-world data obtained from the German Pain e-Registry (GPeR), a nationwide, web-based registry documenting routine pain care in accordance with the legal requirements of the German quality assurance agreement for specialized pain therapy [25].

As with the primary analysis, this secondary evaluation involved a non-interventional, retrospective assessment of anonymized data collected before and during routine clinical care without any study specific patient contact/procedure and did not require approval by an Ethics Committee. The study was conducted in accordance with the principles of the Declaration of Helsinki. The original study protocol, including the planned secondary analyses, was reviewed and approved by the executive board of the German Pain League prior to data mirroring and analysis.

All patients included in the GPeR provided written informed consent for the scientific evaluation of their anonymized data prior to data entry into the registry. The study concept was prospectively registered in the European Medicines Agency (EMA) registry for non-interventional and epidemiological studies (EUPAS identifier: 49718), thereby ensuring transparency and public accessibility of the research objectives.

## 4. Patients and Methods

This secondary analysis was based on depersonalized real-world data derived from the PROVIDENCE study, a retrospective, non-interventional, comparative two-cohort analysis using four-week follow-up data from the German Pain e-Registry (GPeR) [24]. The study design, data collection procedures, ethical considerations, and data protection measures were identical to those described in the primary analysis and complied with the Declaration of Helsinki as well as applicable national and European data protection regulations.

**Figure 1:**
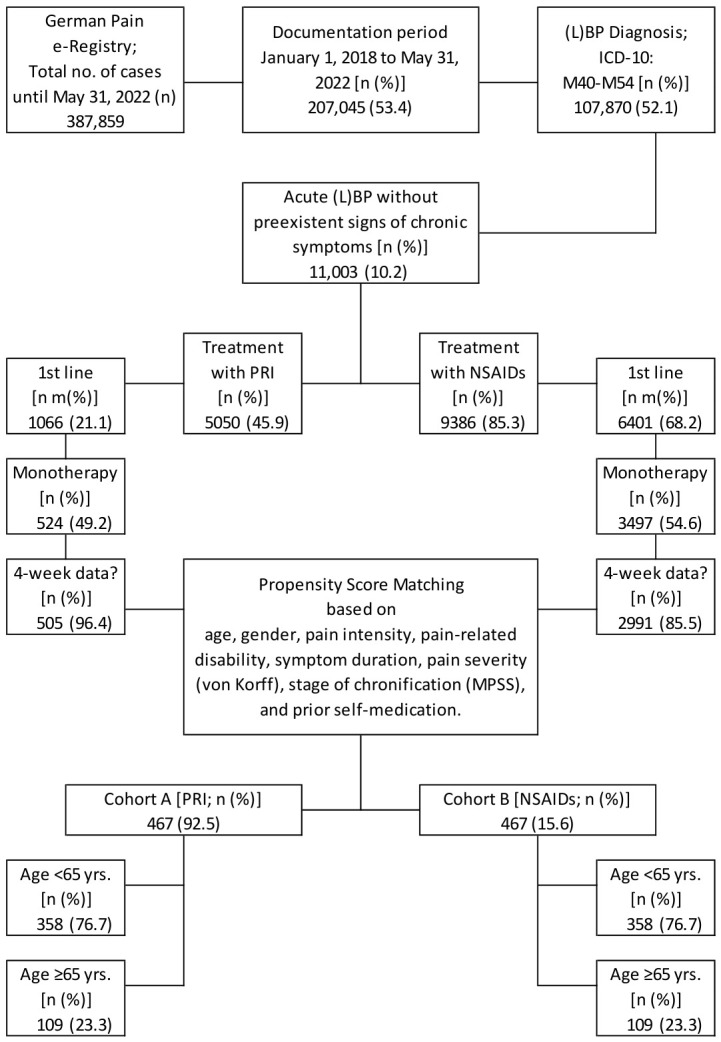
Flow chart of the patient data selection process. Notes: (L)BP: (low) back pain; PRI: pridinol; NSAIDs: nonsteroidal anti-inflammatory drugs; MPSS: Mainz pain staging system.

The study population comprised all patients included in the propensity score-matched cohorts of the original PROVIDENCE analysis (see Figure 1), consisting of 467 patients treated with pridinol and 467 patients treated with NSAIDs [24]. Patients who could not be adequately matched according to predefined propensity score criteria were excluded to optimize baseline comparability and minimize residual confounding. All eligible patients fulfilling predefined inclusion criteria during the observation period were identified from the German Pain e-Registry without selective recruitment procedures. For the purpose of the present secondary analysis, patients were stratified into two predefined age groups, <65 years and ≥65 years. This age threshold was selected because it is widely used in clinical pharmacology to define older populations at increased risk for adverse drug reactions and was explicitly considered in the original dataset for the assessment of NSAID-related clinical and pharmacological risk factors.

Treatment allocation followed routine clinical practice and was not influenced by the study. Patients in the pridinol cohort received pridinol as first-line prescription monotherapy after failure of self-medication or insufficient spontaneous improvement. Patients in the NSAID cohort received NSAID monotherapy, including ibuprofen, diclofenac, naproxen, acetylsalicylic acid, or other approved NSAIDs, according to the treating physician’s judgment. Dosage, treatment duration, and concomitant therapies reflected real-world conditions and adhered to the modified intention-to-treat principle defined in the primary analysis [24].

The primary outcome of this secondary analysis was the incidence of adverse drug reactions (ADRs) during the four-week treatment period, assessed separately for patients younger than 65 years and those aged 65 years or older and compared between treatment groups. Secondary safety outcomes included the incidence of gastrointestinal and/or cardiovascular ADRs, the occurrence of ADR-related treatment discontinuations, and the time to onset of ADRs and ADR-related discontinuation.

Pain-related functional impairment was assessed using a modified version of the original Pain Disability Index (mPDI) [26,27]. The instrument captures impairments in daily activities across seven domains, including home and family responsibilities, recreation, social activities, occupation, self-care or personal maintenance, sleep, and overall quality of life. To ensure harmonization of outcome measures, all items were rated on a 100 mm visual analogue scale (VAS), ranging from 0 (no impairment) to 100 (worst imaginable impairment). Domain scores were aggregated to derive an overall mPDI score, with higher values indicating greater pain-related disability. Clinically meaningful functional improvement was defined a priori as an absolute/relative improvement of at least 20 mm VAS/50% from baseline in the overall mPDI score after four weeks of treatment.

All analyses were performed separately within each age stratum and comparatively between treatment groups. Continuous variables were summarized using means, standard deviations, medians, ranges, and 95% confidence intervals, while categorical variables were described using frequencies and percentages. Between-group comparisons were conducted using the same statistical methods as in the primary analysis, including paired *t*-tests or Wilcoxon signed-rank tests for continuous variables and McNemar or chi-square tests for categorical variables, as appropriate. Effect sizes, odds ratios, relative risks, and numbers needed to treat (NNT) or harm (NNH) were calculated where applicable. All statistical tests were two-sided with a significance level of 0.05. No formal multiplicity adjustment was applied because of the exploratory nature of this predefined secondary analysis. Therefore, statistical findings should be interpreted as hypothesis-generating rather than confirmatory.

## 5. Results

### 5.1. Study Population and Baseline Characteristics

Following propensity score matching, a total of 934 patients with acute (low) back pain were included in the analysis, comprising 467 patients treated with pridinol and 467 patients treated with NSAIDs [ibuprofen n = 351 (73.2%), diclofenac n = 198 (42.4%), acetylsalicylic acid n = 49 (10.5%), naproxen n = 34 (7.3%); concurrent use of multiple medications is possible]. Within each treatment cohort, 358 patients (76.7%) were younger than 65 years and 109 patients (23.3%) were aged 65 years or older. The patient selection process is summarized in Figure 1. Baseline demographic and clinical characteristics were well balanced between treatment groups within each age stratum. No statistically significant between-group differences were observed with respect to age, sex distribution, body mass index, pain severity according to von Korff grading, stage of pain chronification according to the Mainz Pain Staging System, duration of the acute pain episode, or prevalence of relevant comorbidities (Table 1). Clinical and pharmacological risk factors for NSAID use were highly prevalent in both age groups, with all patients aged 65 years or older presenting with at least one documented risk factor.

### 5.2. Occurrence of Adverse Drug Reactions

Across the entire study population, at least one adverse drug reaction (ADR) was reported by 14.9% of patients, with a substantially lower proportion among patients treated with pridinol (9.0%) compared with those treated with NSAIDs (20.8%; *p* < 0.001; see Table 2 and Table 3). When stratified by age, marked differences emerged between treatment groups. In the pridinol cohort, the proportion of patients experiencing at least one ADR was virtually identical in patients younger than 65 years and those aged 65 years or older (8.9% vs. 9.2%; *p* = 0.911). In contrast, NSAID-treated patients showed a pronounced age-related increase in ADR incidence, with 17.3% of patients younger than 65 years and 32.1% of patients aged 65 years or older reporting at least one ADR (*p* < 0.001).

A similar pattern was observed for the occurrence of multiple ADRs. In the pridinol cohort, the proportion of patients experiencing two or more ADRs remained low and comparable across age groups (<65 vs. ≥65 yrs: 1.7 vs. 1.8%; *p* = 0.930), whereas NSAID-treated patients—particularly those aged 65 years or older—showed a substantially higher frequency of multiple ADRs (<65 vs. ≥65 yrs: 4.2 vs. 13.8%; *p* < 0.001).

Age-dependent differences were also evident with regard to ADR-related treatment discontinuations. Overall, ADR-related discontinuation occurred in 3.4% of patients treated with pridinol and in 9.4% of patients treated with NSAIDs (*p* < 0.001). Among patients younger than 65 years, ADR-related discontinuation rates were 3.1% for pridinol and 5.9% for NSAIDs (*p* = 0.071), whereas in patients aged 65 years or older, corresponding rates were 4.6% and 21.1%, respectively (*p* < 0.001). Thus, while ADR-related discontinuation rates under pridinol remained low and largely age independent, a pronounced increase was observed among older patients receiving NSAIDs.

**Figure 2:**
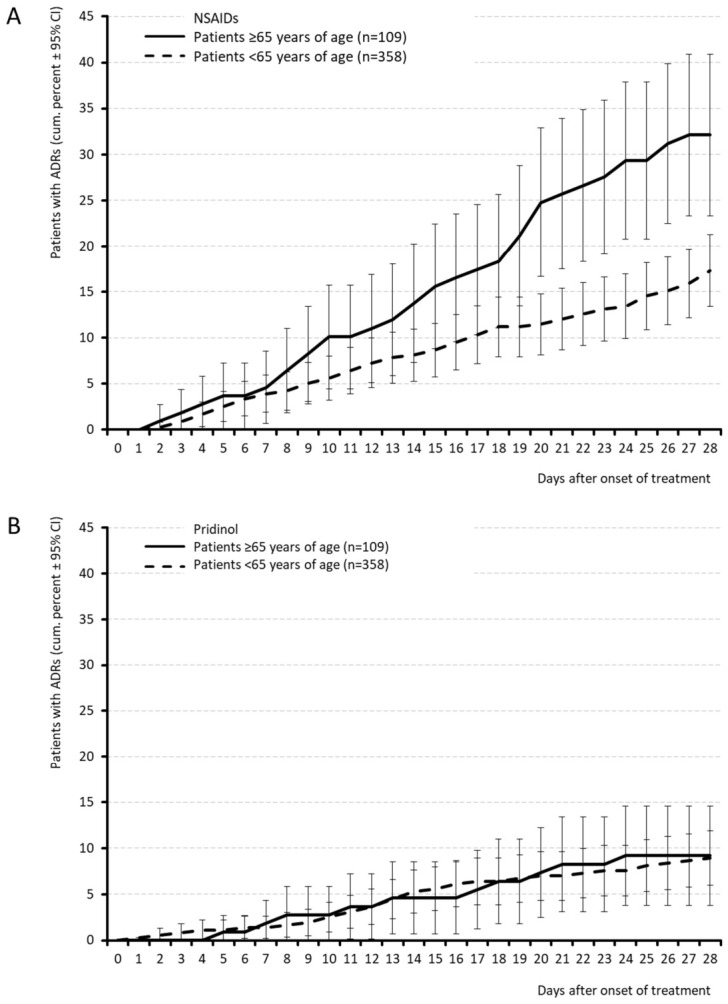
Time to ADR occurrence diagrams for NSAIDs ((**A**), upper panel) and pridinol ((**B**), lower panel). Notes: ADR: adverse drug reaction; NSAIDs: nonsteroidal anti-inflammatory drugs.

**Figure 3:**
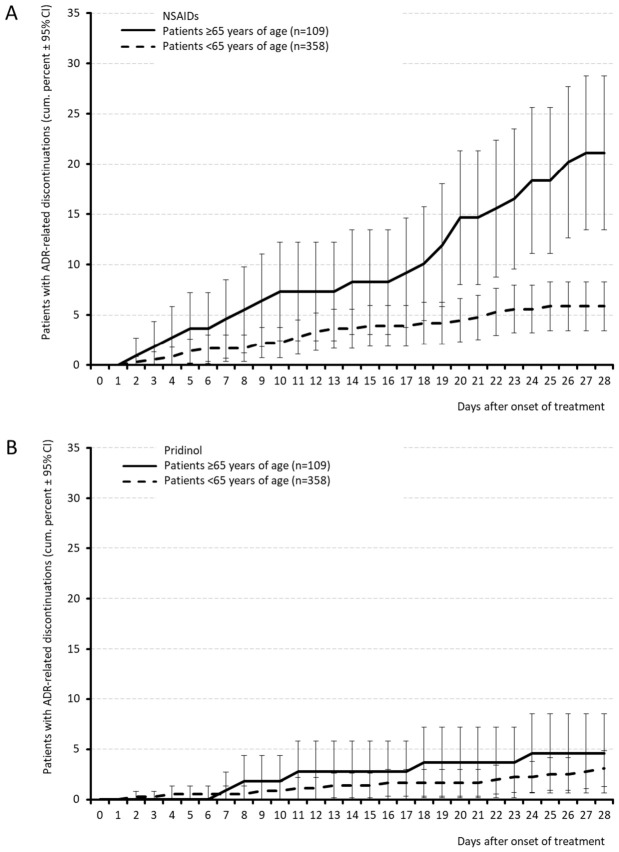
Time to ADR-related treatment discontinuation diagram for NSAIDs ((**A**), upper panel) and pridinol ((**B**), lower panel). Notes: ADR: adverse drug reaction; NSAIDs: nonsteroidal anti-inflammatory drugs.

Time-to-event analyses further supported these findings, demonstrating an earlier onset and a steeper cumulative increase in ADR occurrence among NSAID-treated patients compared with pridinol-treated patients, with this difference being particularly pronounced in the ≥65-year age group (Figure 2). A similar pattern was observed for time to ADR-related treatment discontinuation, with substantially higher and earlier discontinuation rates among older NSAID-treated patients, while cumulative curves for pridinol overlapped across age groups (Figure 3).

### 5.3. Spectrum of Adverse Drug Reactions

The distribution of ADRs differed substantially between treatment groups and across age strata. Gastrointestinal disorders constituted the most frequent category of ADRs overall and were predominantly observed in patients treated with NSAIDs (Table 2). In the overall cohort, gastrointestinal ADRs were reported by 17.6% of NSAID-treated patients compared with 3.6% of pridinol-treated patients (*p* < 0.001). When stratified by age, gastrointestinal ADRs occurred in 14.2% of NSAID-treated patients younger than 65 years and increased to 28.4% in those aged 65 years or older (*p* < 0.001), whereas corresponding rates in the pridinol cohort remained low and comparable between age groups (3.6% and 3.7%, respectively; *p* = 0.985).

These findings were corroborated by analyses focusing on gastrointestinal and/or cardiovascular ADRs (Table 4). Among NSAID-treated patients, the proportion experiencing at least one gastrointestinal or cardiovascular ADR increased markedly with age (from 18.2% for those <65 yrs. to 41.3% for patients 65 yrs; *p* <0.001), whereas no relevant age-related difference was observed in the pridinol cohort (4.7 vs. 5.5%; *p* = 0.749). Between-treatment comparisons within both age strata showed significantly lower rates of gastrointestinal and cardiovascular ADRs under pridinol than under NSAIDs (all *p* < 0.001).

**Figure 4:**
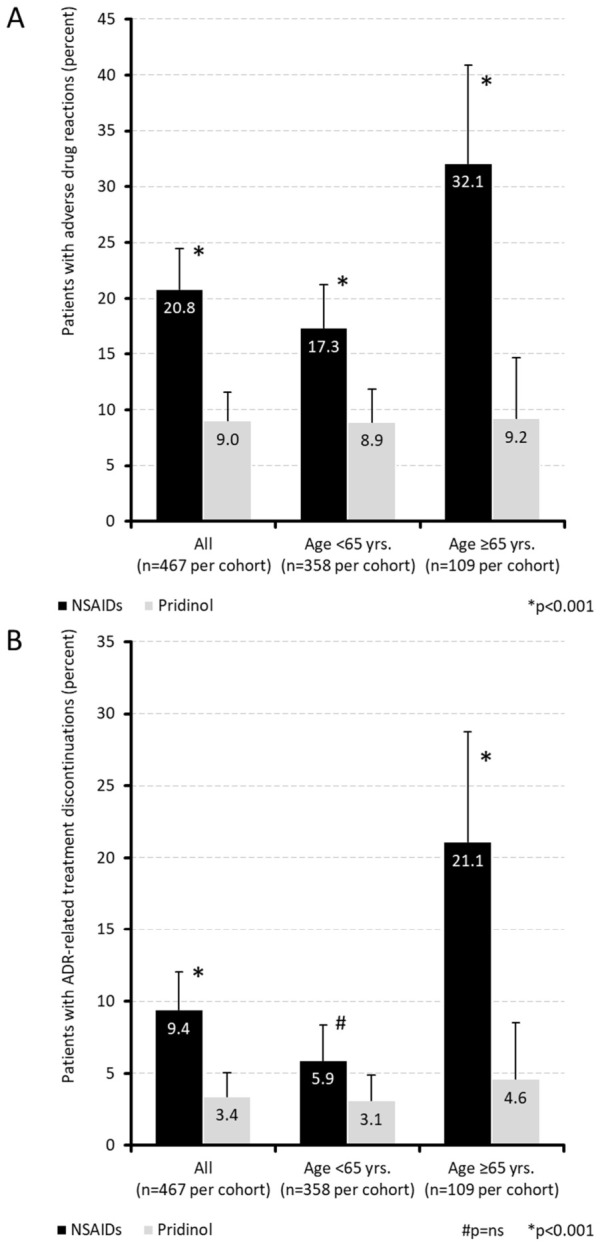
Occurrence of adverse drug reactions (ADRs; (**A**), upper panel) and ADR-related treatment discontinuations ((**B**), lower panel) dependent on treatment type and age.

Adverse reactions affecting the nervous system, such as headache and dizziness, occurred infrequently and at similar rates across treatment groups and age strata, with no statistically significant age-related differences within cohorts (Table 2). Minor anticholinergic-type ADRs, including dry mouth, were rare overall (0.7%) and occurred more frequently in patients with pridinol (1.3%) compared to those with NSAIDs (0.2%; *p* = 0.058), without evidence of a clinically relevant age-related increase. Cardiac and vascular ADRs, including hypertension and peripheral edema, were observed primarily in NSAID-treated patients and were numerically more frequent in older individuals, although absolute event numbers remained low (Table 2 and Table 4). A summary of ADR occurrence and ADR-related treatment discontinuations by treatment type and age is provided in Figure 4.

### 5.4. Functional Outcomes

Clinically meaningful improvement in pain-related functional disability, defined as an absolute/relative reduction of at least 20 mm VAS and/or 50% from baseline in the modified Pain Disability Index, was achieved by a high proportion of patients treated with pridinol across both age groups (Table 5). Improvement rates were 91.9% in patients younger than 65 years and 88.1% in patients aged 65 years or older (*p* = 0.222). In the NSAID cohort, corresponding rates were substantially lower and comparable between age groups, with 48.0% of younger patients and 47.7% of older patients achieving a clinically relevant improvement (*p* = 0.951). Irrespective of age, mPDI improvement rates were significantly higher with PRI compared to those seen with NSAIDs (*p* < 0.001).

## 6. Discussion

This age-stratified secondary analysis of real-world data from the PROVIDENCE study demonstrates that patient age is a key determinant of the safety profile of NSAIDs, but not of pridinol, in the treatment of acute (low) back pain. While the overall superiority of pridinol over NSAIDs with regard to tolerability has already been established in the original primary analysis, the present findings extend this evidence by showing that the excess risk associated with NSAID therapy increases markedly with advancing age, whereas the safety profile of pridinol remains largely unaffected.

A central observation of this analysis is that the pronounced age dependency of NSAID-related adverse drug reactions cannot be sufficiently explained by differences in baseline characteristics or comorbidity burden. Despite a mean age difference of approximately 14 years, younger and older patients were largely comparable with respect to documented comorbidities, clinical and pharmacological NSAID risk factors, pain severity, and stage of chronification. Nevertheless, NSAID-treated patients aged 65 years or older experienced a substantially higher incidence of adverse drug reactions and a more than threefold higher rate of ADR-related treatment discontinuations compared with younger patients. In contrast, ADR incidence and discontinuation rates under pridinol were low and virtually identical across age groups.

These findings strongly suggest that chronological age itself represents a major and clinically independent modifier of NSAID-related risk, beyond the presence of measurable comorbidities. Age-related physiological changes affecting pharmacokinetics and pharmacodynamics, reduced renal and gastrointestinal functional reserve, and diminished compensatory capacity may substantially increase vulnerability to NSAID toxicity in older patients, even when traditional risk factors are comparable. From a clinical perspective, this implies that increasing age—particularly from the age of 65 years onward—should be regarded as a central risk factor when considering NSAID therapy for acute (low) back pain.

The spectrum of adverse drug reactions observed in this analysis further supports this interpretation. Gastrointestinal and cardiovascular adverse reactions were the dominant contributors to the age-related increase in NSAID toxicity and showed a clear age relationship, whereas corresponding events under pridinol remained rare and age independent. Importantly, adverse reactions potentially related to anticholinergic mechanisms, which are often considered a concern in older populations, were infrequent under pridinol and did not increase with age in our analysis. This finding challenges the common perception that anticholinergic antispasmodics necessarily pose a higher risk in older patients and suggests that such concerns may not be justified for pridinol when used at recommended doses.

With regard to effectiveness, high response and improvement rates were observed in both treatment groups, which must be interpreted in the context of the well-known high rate of spontaneous remission in acute (low) back pain. Nevertheless, clinically meaningful improvements in pain-related disability were consistently and substantially more frequent under pridinol than under NSAIDs across both age groups. This difference is plausibly explained by the mechanism-oriented therapeutic approach of pridinol, which directly targets increased muscle tone and muscle spasm—key pathophysiological components of acute (low) back pain—whereas NSAIDs exert a more unspecific anti-inflammatory and analgesic effect. However, given the observational design and the high spontaneous recovery rate characteristic of acute low back pain, differences in functional outcomes should be interpreted cautiously. Expectation effects, differences in adherence, co-interventions, and physician treatment preferences may have contributed to the observed results.

Crucially, the modest effectiveness of NSAIDs was accompanied by a significantly worse tolerability profile, particularly in older patients. Thus, in this population, limited functional benefit appears to be achieved at the expense of a substantially increased risk of adverse drug reactions and premature treatment discontinuation. In contrast, pridinol combined sustained functional improvement with a consistently favorable and age-independent safety profile, resulting in a more balanced benefit–risk relationship, especially in patients aged 65 years or older.

Taken together, these findings highlight the importance of age-adapted and mechanism-oriented pharmacological treatment strategies in acute (low) back pain. They suggest that NSAID therapy should be critically reconsidered in older patients, particularly when safer alternatives with demonstrated effectiveness and age-independent tolerability are available.

## 7. Strengths and Limitations

Strengths of this analysis include the use of a large, well-characterized real-world dataset, rigorous propensity score matching, comprehensive age stratification, and detailed documentation of patient-reported outcomes and adverse drug reactions under routine clinical conditions. The focus on age-dependent treatment effects addresses a clinically highly relevant question that is often insufficiently covered in randomized controlled trials.

Limitations include the retrospective and non-interventional design, which precludes causal inference and may be subject to residual confounding despite matching. Furthermore, multiple exploratory analyses were performed without adjustment for multiplicity. Therefore, findings should be interpreted as hypothesis-generating and considered with appropriate caution. Adverse drug reactions were based on routine documentation and patient reporting and may therefore be influenced by reporting bias. In addition, age-related differences in symptom perception and reporting behavior may have influenced ADR detection. Older patients may report symptoms differently due to increased healthcare utilization or awareness of medication-related effects, whereas mild ADRs may be underreported under routine clinical conditions. The four-week observation period limits conclusions regarding long-term safety and effectiveness. Furthermore, multiple exploratory analyses were performed without adjustment for multiplicity. Therefore, findings should be interpreted as hypothesis-generating and considered with appropriate caution. Finally, as the data were derived from the German healthcare system, generalizability to other healthcare settings may be limited.

## 8. Conclusions

In this age-stratified real-world analysis, patient age emerged as a key determinant of the safety profile of NSAIDs, but not of pridinol, in the treatment of acute (low) back pain. While NSAID therapy was associated with a marked age-related increase in adverse drug reactions and treatment discontinuations, particularly among patients aged 65 years or older, the tolerability of pridinol remained consistently favorable across age groups. At the same time, clinically meaningful improvements in pain-related disability were substantially more frequent under pridinol than under NSAIDs, despite the high rate of spontaneous remission characteristic of acute (low) back pain.

Taken together, these findings suggest that pridinol may provide a favorable benefit–risk profile, particularly in older patients. While potential anticholinergic adverse effects of pridinol should not be disregarded—particularly in vulnerable patient populations—these risks appear to be low and, based on the present data, do not show relevant age dependency. In contrast, the substantially higher and clearly age-related risk associated with NSAID therapy highlights the need for a cautious and critical use of NSAIDs in older and multimorbid patients. Prospective randomized studies, especially in adults aged ≥65 years, are warranted to confirm these findings.

## 9. Key Messages/Clinical Implications

**Patient age is a major determinant of NSAID-related risk in acute (low) back pain**.

NSAID-associated adverse drug reactions and treatment discontinuations increase markedly in patients aged 65 years or older, even when baseline comorbidities and documented risk factors are comparable to those of younger patients.

**Chronological age appears to be an independent risk modifier for NSAIDs.**

The pronounced age dependency of NSAID toxicity cannot be sufficiently explained by measurable comorbidities alone, underscoring the need to consider age itself as a central risk factor.

**Pridinol shows age-independent tolerability.**

In contrast to NSAIDs, the incidence of adverse drug reactions and treatment discontinuations under pridinol remains low and comparable in younger and older patients.

**Effectiveness differences favor mechanism-oriented therapy.**

Despite high spontaneous remission rates in acute (low) back pain, clinically meaningful improvements in pain-related disability were consistently more frequent under pridinol than under NSAIDs across all age groups.

**Clinical implication:**

From the age of 65 years onward, NSAID therapy in acute (low) back pain should be used with particular caution, while mechanism-oriented alternatives such as pridinol may offer a more favorable benefit–risk profile.

## Figures and Tables

**Table 1:** Demographic and baseline characteristics.

	All Patients			Age < 65 Years			Age ≥ 65 Years		
Cohort	A	B	Sign.	A	B	Sign.	A	B	Sign.
Index Medication	PRI	NSAIDs	A vs. B	PRI	NSAIDs	A vs. B	PRI	NSAIDs	A vs. B
Number of Patients	467	467		358	358		109	109	
Age [years; mean (SD)]	56.4 (10.8)		ns *	52.2 (8.3)		ns *	70.2 (4.8)		ns *
(median)	57			54			68		
(range)	28–82			28–64			65–82		
Patients ≥ 65 years [n (%)]	109 (23.3)		ns *	0 (0.0)		ns *	109 (100.0)		ns *
Female gender [n (%)]	259 (55.5)		ns *	194 (54.2)		ns *	65 (59.6)		ns *
ICD-10: M40–43 [n (%)]	38 (8.1)		ns *	24 (6.7)		ns *	14 (12.8)		ns *
M45–49 [n (%)]	58 (12.4)		ns *	40 (11.2)		ns *	18 (16.5)		ns *
M50–54 [n (%)]	371 (79.4)		ns *	294 (82.1)		ns *	77 (70.6)		ns *
Body weight [kg; mean (SD)]	77.5 (16.9)	78.1 (17.3)	ns	77.6 (17.1)	78.4 (17.6)	ns	77.2 (16.3)	76.9 (16.3)	ns
Height [cm; mean (SD)]	170.0 (9.3)	169.6 (9.3)	ns	170.1 (9.5)	169.4 (9.3)	ns	169.6 (8.7)	170.1 (9.3)	ns
BMI [kg/sqm; mean (SD)]	26.7 (5.1)	26.9 (5.5)	ns	26.7 (5.1)	27.1 (5.5)	ns	26.8 (5.0)	26.4 (5.4)	ns
MPSS I [n (%)]	422 (90.4)		ns *	317 (88.5)	319 (89.1)	ns *	105 (96.3)	103 (94.5)	ns *
MPSS II [n (%)]	45 (9.6)		ns *	41 (11.5)	39 (10.9)	ns *	4 (3.7)	6 (5.5)	ns *
Pain severity: von Korff I/II [n (%)]	148 (31.7)		ns *	110 (30.7)	109 (30.5)	ns *	38 (34.9)	39 (35.8)	ns *
von Korff III/IV [n (%)]	319 (68.3)		ns *	248 (69.3)	249 (69.5)	ns *	71 (65.1)	70 (64.2)	ns *
a(L)BP-duration [days; mean (SD)]	13.7 (9.6)	13.8 (9.8)	ns *	14.0 (9.9)	13.9 (10.0)	ns *	12.6 (8.3)	13.2 (9.0)	ns *
Comorbidities: GI tract [n (%)]	125 (26.8)	126 (27.0)	ns	91 (25.4)	97 (27.1)	ns	34 (31.2)	29 (26.6)	ns
Allergies [n (%)]	100 (21.4)	99 (21.2)	ns	75 (20.9)	78 (21.8)	ns	25 (22.9)	21 (19.3)	ns
Cardio-/Vascular system [n (%)]	99 (21.2)	98 (21.0)	ns	73 (20.4)	78 (21.8)	ns	26 (23.9)	20 (18.3)	ns
Pulmonary system [n (%)]	57 (12.2)	56 (12.0)	ns	50 (14.0)	45 (12.6)	ns	7 (6.4)	11 (10.1)	ns
Coagulation system [n (%)]	53 (11.3)	55 (11.8)	ns	42 (11.7)	38 (10.6)	ns	11 (10.1)	17 (15.6)	ns
Renal system [n (%)]	52 (11.1)	49 (10.5)	ns	38 (10.6)	35 (9.8)	ns	14 (12.8)	14 (12.8)	ns
Skin [n (%)]	53 (11.3)	54 (11.6)	ns	44 (12.3)	42 (11.7)	ns	9 (8.3)	12 (11.0)	ns
Hepato-billiary tract [n (%)]	38 (8.1)	40 (8.6)	ns	31 (8.7)	29 (8.1)	ns	7 (6.4)	11 (10.1)	ns
Clinical risk factors for NSAIDS [n (%)]	315 (67.5)	322 (69.0)	ns	239 (66.8)	247 (69.0)	ns	76 (69.7)	75 (68.8)	ns
Pharmacological risk factors for NSAIDs [n (%)]	280 (60.0)	273 (58.5)	ns	211 (58.9)	212 (59.2)	ns	69 (63.3)	61 (56.0)	ns
Any risk factor for NSAIDs [n (%)]	366 (78.4)	371 (79.4)	ns	257 (71.8)	262 (73.2)	ns	109 (100.0)	109 (100.0)	ns

Notes: PRI: pridinol, NSAIDs: nonsteroidal anti-inflammatory drugs; SD: standard deviation; ICD-10: International Classification of Diseases, Version 10; BMI: body mass index; MPSS: Mainz pain staging system; a(L)BP: acute (low) back pains; * refers to those parameters used for the propensity score matching process.

**Table 2:** Summary of age-related adverse drug reactions (ADRs) reported during the 4-week treatment evaluation for a(L)BP patients treated either with PRI or NSAID.

			All Patients						Patients < 65 Years of Age						Patients ≥ 65 Years of Age					
	All (n = 934)		PRI (n = 467)		NSAIDs (n = 467)				PRI (n = 358)		NSAIDs (n = 358)				PRI (n = 109)		NSAIDs (n = 109)			
	n	(% Pats.)	n	(% Pats.)	n	(% Pats.)	Sign.	(ES)	n	(% Pats.)	n	(% Pats.)	Sign.	(ES)	n	(% Pats.)	n	(% Pats.)	Sign.	(ES)
Number of ADRs reported	187	-	51	-	136	-			39	-	83	-			12	-	53	-		
Patients with ≥1 ADR	139	(14.9)	42	(9)	97	(20.8)	<0.001	(0.165)	32	(8.9)	62	(17.3)	0.003	(0.124)	10	(9.2)	35	(32.1)	<0.001	(0.283)
Patients with ≥2 ADRs	38	(4.1)	8	(1.7)	30	(6.4)	<0.001	(0.095)	6	(1.7)	15	(4.2)	<0.001	(0.045)	2	(1.8)	15	(13.8)	<0.001	(0.163)
Patients with ADR-related treatment discontinuation	60	(6.4)	16	(3.4)	44	(9.4)	<0.001	(0.122)	11	(3.1)	21	(5.9)	0.071	(0.068)	5	(4.6)	23	(21.1)	<0.001	(0.247)
Nervous system disorders (SOC)	40	(4.3)	21	(4.5)	19	(4.1)	0.747	(0.011)	15	(4.2)	14	(3.9)	0.850	(0.007)	6	(5.5)	5	(4.6)	0.757	(0.021)
Headache (PT)	29	(3.1)	17	(3.6)	12	(2.6)	0.458	(0.031)	12	(3.4)	9	(2.5)	0.663	(0.025)	5	(4.6)	3	(2.8)	0.724	(0.049)
Dizziness (PT)	11	(1.2)	4	(0.9)	7	(7)	0.546	(0.03)	3	(0.8)	5	(1.4)	0.724	(0.027)	1	(0.9)	2	(1.8)	1.000	(0.039)
Gastrointestinal disorders (SOC)	99	(10.6)	17	(3.6)	82	(17.6)	<0.001	(0.226)	13	(3.6)	51	(14.2)	<0.001	(0.186)	4	(3.7)	31	(28.4)	<0.001	(0.337)
Abdominal pain (PT)	34	(3.6)	5	(1.1)	29	(6.2)	<0.001	(0.137)	3	(0.8)	19	(5.3)	0.001	(0.129)	2	(1.8)	10	(9.2)	0.043	(0.161)
Dyspepsia (PT)	18	(1.9)	0	(0)	18	(3.9)	<0.001	(0.14)	0	(0)	10	(2.8)	0.004	(0.119)	0	(0)	8	(7.3)	0.013	(0.195)
Diarrhoea (PT)	15	(1.6)	0	(0)	15	(3.2)	<0.001	(0.128)	0	(0)	8	(2.2)	0.013	(0.106)	0	(0)	7	(6.4)	0.023	(0.182)
Nausea (PT)	15	(1.6)	6	(1.3)	9	(1.9)	0.606	(0.026)	6	(1.7)	5	(1.4)	1.000	(0.011)	0	(0)	4	(3.7)	0.134	(0.137)
Vomiting (PT)	10	(1.1)	0	(0)	10	(2.1)	0.004	(0.104)	0	(0)	8	(2.2)	0.013	(0.106)	0	(0)	2	(1.8)	0.480	(0.096)
Dry mouth (PT)	7	(0.7)	6	(1.3)	1	(0.2)	0.131	(0.062)	4	(1.1)	1	(0.3)	0.371	(0.05)	2	(1.8)	0	(0)	0.480	(0.096)
General disorders and administration site conditions (SOC)	14	(1.5)	7	(1.5)	7	(1.5)	1.000	(0)	7	(2)	3	(0.8)	0.203	(0.048)	0	(0)	4	(3.7)	0.044	(0.137)
Oedema peripheral (PT)	6	(0.6)	0	(0)	6	(1.3)	0.041	(0.08)	0	(0)	3	(0.8)	0.248	(0.065)	0	(0)	3	(2.8)	0.248	(0.118)
Fatigue (PT)	5	(0.5)	4	(0.9)	1	(0.2)	0.371	(0.044)	4	(1.1)	0	(0)	0.134	(0.075)	0	(0)	1	(0.9)	1.000	(0.068)
Asthenia (PT)	3	(0.3)	3	(0.6)	0	(0)	0.248	(0.057)	3	(0.8)	0	(0)	0.248	(0.065)	0	(0)	0	(0)	na	na
Vascular disorders (SOC)	11	(1.2)	2	(0.4)	9	(1.9)	0.034	(0.069)	2	(0.6)	6	(1.7)	0.155	(0.053)	0	(0)	3	(2.8)	0.081	(0.118)
Hypertension (PT)	9	(1)	0	(0)	9	(1.9)	0.008	(0.099)	0	(0)	6	(1.7)	0.041	(0.092)	0	(0)	3	(2.8)	0.081	(0.118)
Hypotension (PT)	2	(0.2)	2	(0.4)	0	(0)	0.480	(0.046)	2	(0.6)	0	(0)	0.480	(0.053)	0	(0)	0	(0)	na	na
Cardiac disorders (SOC)	9	(1)	4	(0.9)	5	(1.1)	0.738	(0.011)	2	(0.6)	0	(0)	0.157	(0.053)	2	(1.8)	5	(4.6)	0.249	(0.078)
Tachycardia (PT)	5	(0.5)	1	(0.2)	4	(0.9)	0.371	(0.044)	1	(0.3)	0	(0)	1.000	(0.037)	0	(0)	4	(3.7)	0.134	(0.137)
Circulatory collapse (PT)	4	(0.4)	3	(0.6)	1	(0.2)	0.617	(0.033)	1	(0.3)	0	(0)	1.000	(0.037)	2	(1.8)	1	(0.9)	1.000	(0.039)
Metabolism and nutrition disorders (SOC)	8	(0.9)	0	(0)	8	(1.7)	0.005	(0.093)	0	(0)	5	(1.4)	0.025	(0.084)	0	(0)	3	(2.8)	0.081	(0.118)
Decreased appetite (PT)	8	(0.9)	0	(0)	8	(1.7)	0.013	(0.093)	0	(0)	5	(1.4)	0.074	(0.084)	0	(0)	3	(2.8)	0.248	(0.118)
Skin and subcutaneous tissue disorders (SOC)	5	(0.5)	0	(0)	5	(1.1)	0.074	(0.073)	0	(0)	4	(1.1)	0.045	(0.075)	0	(0)	1	(0.9)	0.316	(0.068)
Rash (PT)	5	(0.5)	0	(0)	5	(1.1)	0.074	(0.073)	0	(0)	4	(1.1)	0.134	(0.075)	0	(0)	1	(0.9)	1.000	(0.068)
Psychiatric disorders (SOC)	1	(0.1)	0	(0)	1	(0.2)	0.317	(0.033)	0	(0)	0	(0)	na	na	0	(0)	1	(0.9)	0.316	(0.068)
Restlessness (PT)	1	(0.1)	0	(0)	1	(0.2)	0.317	(0.033)	0	(0)	0	(0)	na	na	0	(0)	1	(0.9)	0.316	(0.068)

Notes: ADRs: adverse drug reactions.

**Table 3:** Occurrence of adverse drug reactions (ADRs) dependent on treatment type and age.

Patients with ≥1 ADR	A: Pridinol		B: NSAIDs		Sign (A vs. B)	ES	OR A vs. B (95% CI)	OR B vs. A (95% CI)	NNH
All (n = 467)	42	(9.0)	97	(20.8)	<0.001	0.165	0.38 (0.31–0.61)	2.65 (1.80–3.91)	8
[n (a%)]									
C: Age < 65 yrs. (n = 358)	32	(8.9)	62	(17.3)	<0.001	0.124	0.47 (0.30–0.74)	2.13 (1.35–3.36)	12
[n (a%)]									
D: Age ≥ 65 yrs. (n = 109)	10	(9.2)	35	(32.1)	<0.001	0.283	0.21 (0.10–0.46)	4.68 (2.18–10.06)	4
[n (a%)]									
Significance (C vs. D)	0.940		<0.001						
Effect size (ES)	0.003		0.154						
Odds ratio C vs. D	0.97		0.44						
(95% CI)	(0.46–2.05)		(0.27–0.72)						
Odds ratio D vs. C	1.03		2.26						
(95% CI)	(0.52–2.17)		(1.39–3.67)						
NNH	424		7						

**Table 4:** Occurrence of gastrointestinal (GI) and/or cardiovascular (CV) adverse drug reactions (ADRs) dependent on treatment type and age.

Patients with ≥1 GI/CV-ADR	A: Pridinol		B: NSAIDs		Sign (A vs. B)	ES	OR A vs. B (95% CI)	OR B vs. A (95% CI)	NNH
All (n = 467)	23	(4.9)	110	(23.6)	<0.001	0.267	0.17 (0.11–0.27)	5.95 (3.72–9.52)	5
[n (a%)]									
C: Age < 65 yrs. (n = 358)	17	(4.7)	65	(18.2)	<0.01	0.211	0.23 (0.13–0.39)	4.45 (2.55–7.76)	7
[n (a%)]									
D: Age ≥ 65 yrs. (n = 109)	6	(5.5)	45	(41.3)	<0.001	0.423	0.08 (0.03–0.21)	12.07 (4.87–29.90)	3
[n (a%)]									
Significance (C vs. D)	0.749		<0.001						
Effect size (ES)	0.015		0.231						
Odds ratio C vs. D	0.86		0.32						
(95% CI)	(0.33–2.23)		(0.20–0.50)						
Odds ratio D vs. C	1.17		3.17						
(95% CI)	(0.45–3.04)		(1.99–5.05)						
NNH	132		4						

**Table 5:** Improvement of (L)BP-related disability dependent on treatment type and age.

Patients with ≥50% mPDI								
Improvement vs. BL	A: Pridinol		B: NSAIDs		Sign (A vs. B)	ES	OR A vs. B (95% CI)	NNT
All (n = 467)	425	(91.0)	224	(48.0)	<0.001	0.467	10.98 (7.62–15.82)	2
[n (a%)]								
C: Age < 65 yrs. (n = 358)	329	(91.9)	172	(48.0)	<0.001	0.478	12.27 (7.96–18.91)	2
[n (a%)]								
D: Age ≥ 65 yrs. (n = 109)	96	(88.1)	52	(47.7)	<0.001	0.432	8.10 (4.06–16.15)	2
[n (a%)]								
Significance (C vs. D)	0.222		0.951					
Effect size (ES)	0.057		0.003					
Odds ratio C vs. D	1.54		1.01					
(95% CI)	(0.77–3.07)		(0.66–1.56)					
NNT	26		296					

## Data Availability

The original contributions presented in this study are included in the article. Further inquiries can be directed to the corresponding author.

## References

[1] Hartvigsen J., Hancock M.J., Kongsted A., Louw Q., Ferreira M.L., Genevay S., Hoy D., Karppinen J., Pransky G., Sieper J. (2018). What low back pain is and why we need to pay attention. Lancet.

[2] Maher C., Underwood M., Buchbinder R. (2017). Non-specific low back pain. Lancet.

[3] Hoy D., Bain C., Williams G., March L., Brooks P., Blyth F., Woolf A., Vos T., Buchbinder R. (2012). A systematic review of the global prevalence of low back pain. Arthritis Rheum..

[4] Henschke N., Maher C.G., Refshauge K.M., Herbert R.D., Cumming R.G., Bleasel J., York J., Das A., McAuley J.H. (2008). Prognosis in patients with recent onset low back pain in Australian primary care: Inception cohort study. BMJ.

[5] Itz C.J., Geurts J.W., van Kleef M., Nelemans P. (2013). Clinical course of non-specific low back pain: A systematic review of prospective cohort studies set in primary care. Eur. J. Pain.

[6] Pengel L.H., Herbert R.D., Maher C.G., Refshauge K.M. (2003). Acute low back pain: Systematic review of its prognosis. BMJ.

[7] Von Korff M., Saunders K. (1996). The course of back pain in primary care. Spine.

[8] GBD 2019 Diseases and Injuries Collaborators (2020). Global burden of 369 diseases and injuries in 204 countries and territories, 1990–2019: A systematic analysis for the Global Burden of Disease Study 2019. Lancet.

[9] Qaseem A., Wilt T.J., McLean R.M., Forciea M.A., Denberg T.D., Barry M.J., Boyd C., Chow R.D., Fitterman N., Clinical Guidelines Committee of the American College of Physicians (2017). Noninvasive Treatments for Acute, Subacute, and Chronic Low Back Pain: A Clinical Practice Guideline From the American College of Physicians. Ann. Intern. Med..

[10] (2017). Nationale VersorgungsLeitlinie Kreuzschmerz.

[11] Enthoven W.T., Roelofs P.D., Deyo R.A., van Tulder M.W., Koes B.W. (2016). Non-steroidal anti-inflammatory drugs for chronic low back pain. Cochrane Database Syst. Rev..

[12] Bally M., Dendukuri N., Rich B., Nadeau L., Helin-Salmivaara A., Garbe E., Brophy J.M. (2017). Risk of acute myocardial infarction with NSAIDs in real world use: Bayesian meta-analysis of individual patient data. BMJ.

[13] Lanas A., Chan F.K.L. (2017). Peptic ulcer disease. Lancet.

[14] Ungprasert P., Cheungpasitporn W., Crowson C.S., Matteson E.L. (2015). Individual non-steroidal anti-inflammatory drugs and risk of acute kidney injury: A systematic review and meta-analysis of observational studies. Eur. J. Intern. Med..

[15] van Dieën J.H., Reeves N.P., Kawchuk G., van Dillen L.R., Hodges P.W. (2019). Motor Control Changes in Low Back Pain: Divergence in Presentations and Mechanisms. J. Orthop. Sports Phys. Ther..

[16] European Medicines Agency (2018). Pridinol Product Information.

[17] Sweetman S.C. (2020). Martindale: The Complete Drug Reference.

[18] Campbell N.L., Boustani M.A., Lane K.A., Gao S., Hendrie H., Khan B.A., Murrell J.R., Unverzagt F.W., Hake A., Smith-Gamble V. (2010). Use of anticholinergics and the risk of cognitive impairment in an African American population. Neurology.

[19] Salahudeen M.S., Duffull S.B., Nishtala P.S. (2015). Anticholinergic burden quantified by anticholinergic risk scales and adverse outcomes in older people: A systematic review. BMC Geriatr..

[20] Überall M.A., Essner U., Müller-Schwefe G.H.H. (2022). Efficacy and safety/tolerability of pridinol: A meta-analysis of double-blind, randomized, placebo-controlled trials in adult patients with muscle pain. Curr. Med. Res. Opin..

[21] Überall M.A., Müller-Schwefe G.H.H., Horlemann J. (2022). Efficacy and tolerability of the antispasmodic, pridinol, in patients with muscle-pain—Results of primepain, a retrospective analysis of open-label real-world data provided by the German pain E-registry. Curr. Med. Res. Opin..

[22] Cashin A.G., Wand B.M., O’Connell N.E., Lee H., Rizzo R.R., Bagg M.K., O’Hagan E., Maher C.G., Furlan A.D., van Tulder M.W. (2023). Pharmacological treatments for low back pain in adults: An overview of Cochrane Reviews. Cochrane Database Syst. Rev..

[23] Ramos H., Moreno L., Pérez-Tur J., Cháfer-Pericás C., García-Lluch G., Pardo J. (2022). CRIDECO Anticholinergic Load Scale: An Updated Anticholinergic Burden Scale. Comparison with the ACB Scale in Spanish Individuals with Subjective Memory Complaints. J. Pers. Med..

[24] Überall M.A., Schikowski A. (2024). Effectiveness of the Antispasmodic Pridinol vs. Nsaids in Patients with Acute (Low) Back Pain—Results of Providence, a Retrospective, Non-Interventional Propensity- Score Matched Dual Cohort Analysis of Depersonalized 4-Week Real-World Data Provided by The German Pain E-Registry. Arch. Pharmacol. Res..

[25] The National Association of Statutory Health Insurance Physicians and the Regional Associations of Statutory Health Insurance Physicians Agreement on Quality Assurance Measures According to § 135 Para. 2 SGB V for Pain Therapy Care of Patients with Chronic Pain (German: Vereinbarung von Qualitätssicherungsmaßnahmen nach § 135 Abs. 2 SGB V zur Schmerztherapeutischen Versorgung Chronisch Schmerzkranker Patienten). https://www.kbv.de/documents/infothek/rechtsquellen/bundesmantelvertrag/anlage-03-qualitaetssicherung/qs-vereinbarung-schmerztherapie.pdf.

[26] Tait R.C., Chibnall J.T., Krause S. (1990). The Pain Disability Index: Psychometric properties. Pain.

[27] German Pain Association Manual for the German Pain Questionnaire. https://www.schmerzgesellschaft.de/fileadmin/pdf/DSF_Handbuch_2020.pdf.

